# Serum phosphate levels modify the impact of parathyroid hormone levels on renal outcomes in kidney transplant recipients

**DOI:** 10.1038/s41598-020-70709-4

**Published:** 2020-08-13

**Authors:** Yohei Doi, Takayuki Hamano, Naotsugu Ichimaru, Kodo Tomida, Yoshitsugu Obi, Naohiko Fujii, Satoshi Yamaguchi, Tatsufumi Oka, Yusuke Sakaguchi, Isao Matsui, Jun-ya Kaimori, Toyofumi Abe, Ryoichi Imamura, Shiro Takahara, Yoshiharu Tsubakihara, Norio Nonomura, Yoshitaka Isaka

**Affiliations:** 1 Department of Nephrology, Osaka University Graduate School of Medicine, Suita, Japan; 2 Department of Inter-Organ Communication Research in Kidney Disease, Osaka University Graduate School of Medicine, Suita, Japan; 3 Department of Nephrology, Nagoya City University Graduate School of Medical Sciences, 1-Kawasumi, Mizuho-cho, Mizuho-ku, Nagoya, 467-8601 Japan; 4 Department of Advanced Technology for Transplantation, Osaka University Graduate School of Medicine, Suita, Japan; 5 Department of Nephrology, Yodogawa Christian Hospital, Osaka, Japan; 6 Division of Nephrology, University of Tennessee Health Science Center, Memphis, USA; 7 Department of Nephrology, Hyogo Prefectural Nishinomiya Hospital, Nishinomiya, Japan; 8 Department of Urology, Osaka University Graduate School of Medicine, Suita, Japan; 9 Department of Renal Transplantation, Kansai Medical Hospital, Toyonaka, Japan; 10 Graduate School of Health Care Sciences, Jikei Institute, Osaka, Japan

**Keywords:** Nephrology, Renal replacement therapy

## Abstract

Separate assessment of mineral bone disorder (MBD) parameters including calcium, phosphate, parathyroid hormone (PTH), fibroblast growth factor 23 (FGF23), 25-hydroxyvitamin D, and 1,25-dihydroxyvitamin D (1,25D) predict renal outcomes in kidney transplant recipients (KTRs), with conflicting results. To date, data simultaneously evaluating these parameters and interwoven relations on renal outcomes are scarce. We conducted a prospective long-term follow-up cohort study included 263 KTRs with grafts functioning at least 1 year after transplantation. The outcome was a composite of estimated GFR halving and graft loss. Cox regression analyses were employed to evaluate associations between a panel of six MBD parameters and renal outcomes. The outcome occurred in 98 KTRs during a median follow-up of 10.7 years. In a multivariate Cox analysis, intact PTH (iPTH), phosphate, and 1,25D levels were associated with the outcome (hazard ratio, 1.60 per log scale; 95% confidence interval, 1.19–2.14, 1.60 per mg/dL; 1.14–2.23 and 0.82 per 10 pg/mL; 0.68–0.99, respectively). Competing risk analysis with death as a competing event yielded a similar result. After stratification into four groups by iPTH and phosphate medians, high risks associated with high iPTH was not observed in KTRs with low phosphate levels (P-interaction < 0.1). Only KTRs not receiving active vitamin D, poor 1,25D status predicted the worse outcome (P-interaction < 0.1). High iPTH, phosphate, and low 1,25D, but not FGF23, levels predicted poor renal outcomes. Simultaneous evaluation of PTH and phosphate levels may provide additional information regarding renal allograft prognosis.

## Introduction

Kidney transplantation (KTx) is the preferred renal replacement therapy in terms of cost, quality of life, and mortality when compared with maintenance dialysis^1–3^. Over the past decades, short-term graft survival has been improved by the introduction of new immunosuppressants (e.g., calcineurin inhibitors and antimetabolic agents), which help to prevent acute rejection; nonetheless, long-term graft failure remains a major concern^4^. The kidney transplant community needs to identify modifiable factors to further improve longer-term graft survival.

Chronic kidney disease (CKD)–mineral bone disorder (MBD) is a common complication in patients with CKD, and it affects cardiovascular morbidity and mortality^5^. Calcium, phosphate, parathyroid hormone (PTH), fibroblast growth factor 23 (FGF23), 25-hydroxyvitamin D (25D), and 1,25-dihydroxyvitamin D (1,25D) are crucial biochemical components in CKD-MBD. A successful KTx will dramatically improve these laboratory values. However, novel and complex pathophysiology of MBD unique to kidney transplant recipients (KTRs) may occur in post-transplant MBD, which includes the effects of transplant-related drugs, carryover of MBD from the pre-transplant period, and de novo MBD^6^. Recent studies revealed that, when assessed separately, post-transplant MBD parameters were associated with graft prognosis, with conflicting results^7–15^. Since a change in one MBD parameter will affect the others, simultaneous evaluation of MBD parameters is reasonable. Additionally, it is useful to employ these parameters as a panel, if any, combined effects are there in graft outcomes.

To date, there remains a paucity of data simultaneously evaluating MBD parameters and their interwoven relationships on renal outcomes in KTRs. In the current study, we measured a panel of six parameters (calcium, phosphate, PTH, FGF23, 25D, and 1,25D,) among KTRs with grafts functioning at least 1 year after KTx. The aim of this study was twofold: (1) to clarify which MBD parameters predict renal outcomes independent of other variables and (2) to assess the impacts of the interplay among MBD parameters on renal outcomes in a long-term follow-up cohort of KTRs.

## Materials and methods

### Study design and population

We conducted a single-center, prospective observational cohort study (USE-PTH; ultrasonographical evaluation of parathyroid hypertrophy study) at Inoue Hospital, Osaka, Japan. Details of the cohort were published elsewhere^7,16,17^. Briefly, stable KTRs were enrolled in the outpatient Kidney Transplantation Department from August 2007 to May 2008. In this study, we included patients with grafts functioning at least 1 year after KTx in order to avoid drastic changes in MBD parameters following KTx. The study was approved by the Ethics Committee of Inoue Hospital (approval number 179) and was conducted in accordance with the Declaration of Helsinki. All participants provided written informed consent.

### Baseline characteristics and laboratory parameters

Relevant demographics, anthropometrics, laboratory data, medications, and past medical history were extracted from medical records. Transplant-related information, including transplant vintage, donor characteristics (age at transplantation and living or cadaveric donor), immunologic factors (ABO blood-type compatibility and human leukocyte antigen-matching status), and immunosuppressants was also collected. No KTRs received rapamycin, everolimus, or cinacalcet. Nutritional vitamin D (cholecalciferol and ergocalciferol) was not available in Japan. Calcium carbonate was the only phosphate binder available for non-dialysis CKD at the enrollment period.

Blood and urine samples were obtained at enrollment, and blood samples were stored at − 80 °C until analyses. Chemical parameters were measured using standard automated techniques. Urinary protein was assessed via a dipstick test. Serum intact PTH (iPTH) was measured via immunochemiluminometric assay (ECLusys PTH; Roche Diagnostics). Serum 1,25D and 25D levels were assessed by radioimmunoassay (1,25-Dihydroxy Vitamin D RIA Kit; Immunodiagnostic Systems Ltd.) and chemiluminescent immunoassay (The LIAISON 25 OH Vitamin D TOTAL Assay; DiaSorin Inc.), respectively. The biologically active form of FGF23 was evaluated by a sandwich enzyme-linked immunosorbent assay system (Kainos Laboratories, Inc.). The estimated glomerular filtration rate (eGFR) was calculated according to a standard Japanese formula: 194 × creatinine^1.094^ × age^−0.287^ (× 0.739 if female)^18^. Serum Ca levels were corrected on the basis of the following formula: corrected Ca = total Ca + (4.0 − Alb) × 0.8 (in patients with Alb < 4.0 g/dL)^19^. TmP/GFR (tubular maximum reabsorption rate of phosphate to the glomerular filtration rate) was calculated using the following formula^20^: TRP: tubular reabsorption of phosphate = 1 − (U_phosphate_/P_phosphate_) ÷ (U_creatinine_/P_creatinine_) TmP/GFR = TRP × P (if TRP ≤ 0.86), TmP/GFR = 0.3 × TRP × P/ [1 − (0.8 × TRP)] (if TRP > 0.86).

### Outcome

The primary outcome was a composite of eGFR halving and graft loss. Graft loss was defined as the need for dialysis or kidney re-transplantation. Patients were regularly followed up (monthly) at the outpatient clinic until the end of observation (March 31, 2018), lost to follow-up, or dead.

### Statistical analyses

Continuous and categorical variables are expressed as the median (interquartile range) and number (proportion), respectively. We performed Cox regression analyses to assess associations between MBD parameters and primary outcome. The natural log transformation was employed for variables with skewed distribution. A multivariable Cox model was adjusted for a panel of six MBD parameters (calcium, phosphate, iPTH, FGF23, 25D, and 1,25D), case mix (age and gender), graft status (eGFR and proteinuria), transplant-related factors (donor age, donor type, transplant vintage), and use of active vitamin D compounds. We selected the covariates in the multivariable model on the basis of clinical knowledge and previous studies. Proportional hazard assumption was checked using Schoenfeld residuals. Anticipating non-linear associations with the outcome, MBD parameters were parameterized using a restricted cubic spline (RCS) with three knots. We explored potential interactions among significant MBD parameters and the use of active D compounds by including interaction terms in the multivariate Cox model. A competing risk analysis was performed to correctly estimate the probability of renal outcomes in the presence of death as a competing event^21^. The statistical test was two-tailed, and P < 0.05 was considered statistically significant, whereas P < 0.1 was set to indicate statistical significance for interactions^22^. All statistical analyses were performed using the Stata/SE (version 15) software package (StataCorp, College Station, TX).

## Results

### Characteristics of participants

Table 1 indicates the baseline characteristics for the current study. The median age was 50 years, and 62% of participants were male. The graft function was moderately impaired with a median eGFR of 38 mL/min per 1.73 m^2^. Half of participants had transplant vintage of more than 10 years. The bulk of KTRs (> 90%) received standard maintenance immunosuppressive therapy consisting of corticosteroids, calcineurin inhibitors, and antimetabolic agents. With regard to MBD parameters, corrected calcium levels were normal and phosphate levels were within low-normal ranges. Serum iPTH and FGF23 levels were slightly elevated. According to the National Kidney Foundation Kidney.

**Table 1:** Patient characteristics (n = 263).

	Values
**Basic information**	
Age, years	50 (40–59)
Male sex, n (%)	163 (62)
BMI, kg/m^2^	21 (19–24)
SBP, mmHg	121 (114–130)
Prior PTx, n (%)	15 (6)
Dialysis vintage, years	2.3 (1.0–5.7)
Transplant vintage, years	10.7 (4.7–17.2)
ABO incompatibility, n (%)	
Compatible	204 (78)
Incompatible	38 (14)
No information	21 (8)
HLA mismatches (A + B + DR), n (%)	
0	25 (10)
1–3	180 (68)
4–6	23 (9)
No information	35 (13)
Living donor, n (%)	217 (83)
Donor age, years	52 (42–60)
**Medications, n (%)**	
Calcineurin inhibitor	
Cyclosporine	143 (54)
Tacrolimus	97 (37)
None	23 (9)
Antimetabolic agent	
Azathioprine	51 (19)
Mycophenolate mofetil	143 (54)
Mizoribine	46 (18)
None	23 (9)
Prednisolone, n (%)	256 (97)
Active vitamin D compounds, n (%)	116 (44)
CaCO_3_, n (%)	4 (2)
RAAS inhibitors, n (%)	118 (72)
Primary renal disease, n (%)	
Chronic glomerulonephritis	151 (57)
Diabetic nephropathy	13 (5)
Unknown	57 (22)
Others	42 (16)
Laboratory data	
Hemoglobin, g/dL	12.2 (11.2–13.4)
Albumin, mg/dL	4.3 (4.1–4.5)
eGFR, mL/min per 1.73 m^2^	38 (29–52)
Corrected calcium, mg/dL	9.2 (8.9–9.6)
Phosphate, mg/dL	3.1 (2.8–3.6)
25D, ng/mL	16 (13–21)
1,25D, pg/mL	38 (27–51)
Intact PTH, pg/mL	68 (48–101)
Intact FGF23, pg/mL	63 (42–115)
Urinary protein, n (%)	
–	137 (52)
1 +	99 (38)
2–3 +	27 (10)
TmP/GFR, mg/dL	2.3 (1.9–2.7)

The values are presented as median (interquartile range) or n (%).

*BMI* body mass index; *SBP* systolic blood pressure; *PTx* parathyroidectomy; *RAAS* renin–angiotensin–aldosterone system; *eGFR* estimated glomerular filtration rate; *25D* 25-hydroxyvitamin D; *1,25D* 1,25-dihydroxyvitamin D; *PTH* parathyroid hormone; *FGF23* fibroblast growth factor 23; *TmP/GFR* tubular maximum reabsorption rate of phosphate to the glomerular filtration rate.

Disease Outcomes Quality Initiative Guidelines^23^, 96% of our patients were vitamin D insufficient (< 30 ng/mL). Active vitamin D compounds were administered to 44% of participants for indications of hyperparathyroidism and/or osteoporosis. Among these, 58% and 42% were calcitriol (median dose 0.5, interquartile range 0.5–0.5 µg/day) and alfacalcidol (median dose 0.5, interquartile range 0.25–1.0 µg/day) users, respectively. Median 1,25D levels with or without active vitamin D were 31 and 43 pg/mL, respectively. Few participants (2%) received a calcium carbonate.

### MBD parameters and renal outcome

During a median follow-up of 10.7 years, the primary outcome occurred in 98 KTRs (eGFR halving [n = 89] and graft loss [n = 61]), and 22 patients died with a functioning graft. In univariate Cox models, iPTH (hazard ratio [HR] per unit of log iPTH, 1.79; 95% confidence interval [CI] 1.30–2.48), FGF23 (HR per unit of log FGF23, 1.64; 95% CI 1.35–1.99), and phosphate levels (HR per 1 mg/dL 2.49; 95% CI 1.90–3.25) were positively associated with poor renal outcome, whereas 1,25D (HR per 10 pg/mL, 0.68; 95% CI 0.59–0.79) and 25D (HR per 10 ng/mL, 0.71; 95% CI 0.51–0.97) were negatively associated with poor renal outcome (Table 2). On the other hand, corrected calcium levels did not predict any outcome (HR per 1 mg/dL 0.83; 95% CI 0.57–1.21).

**Table 2:** Results of univariable and multivariable Cox models.

Variables	Univariable Cox proportional model			Multivariable Cox proportional model		
	Hazard ratio	95% CI	P value	Hazard Ratio	95% CI	P value
Age, years	1.01	0.99–1.02	0.49	0.99	0.97–1.01	0.36
Male sex	0.83	0.56–1.24	0.37	1.17	0.71–1.91	0.54
eGFR, mL/min/1.73m^2^	0.95	0.93–0.97	< 0.01	0.99	0.97–1.01	0.33
Urine protein -	ref			ref		
1 +	3.48	2.19–5.53	< 0.01	2.85	1.72–4.74	< 0.01
2–3 +	6.02	3.27–11.1	< 0.01	3.54	1.74–7.18	< 0.01
Corrected calcium, mg/dL	0.83	0.57–1.21	0.33	1.13	0.74–1.74	0.56
Phosphate, mg/dL	2.49	1.90–3.25	< 0.01	1.60	1.14–2.23	0.01
25D, 10 ng/mL	0.71	0.51–0-97	0.03	0.80	0.54–1.19	0.27
1.25D, 10 pg/mL	0.68	0.59–0.79	< 0.01	0.82	0.68–0.99	0.04
Log intact PTH	1.79	1.30–2.48	< 0.01	1.60	1.19–2.14	< 0.01
Log intact FGF23	1.64	1.35–1.99	< 0.01	0.99	0.74–1.34	0.97
Active vitamin D compounds	1.27	0.85–1.89	0.24	0.91	0.55–1.49	0.70
Living donor	0.84	0.50–1.42	0.52	0.99	0.52–1.89	0.97
Donor age, year	1.00	0.98–1.01	0.72	1.00	0.98–1.02	0.77
Log transplant vintage	1.55	1.18–2.03	< 0.01	1.30	0.95–1.77	0.10

*95% CI* 95% confidence interval; *GFR* estimated glomerular filtration rate; *25D* 25-hydroxyvitamin D; *1,25D* 1,25-dihydroxyvitamin D; *PTH* parathyroid hormone; *FGF23* fibroblast growth factor 23.

**Figure 1:**
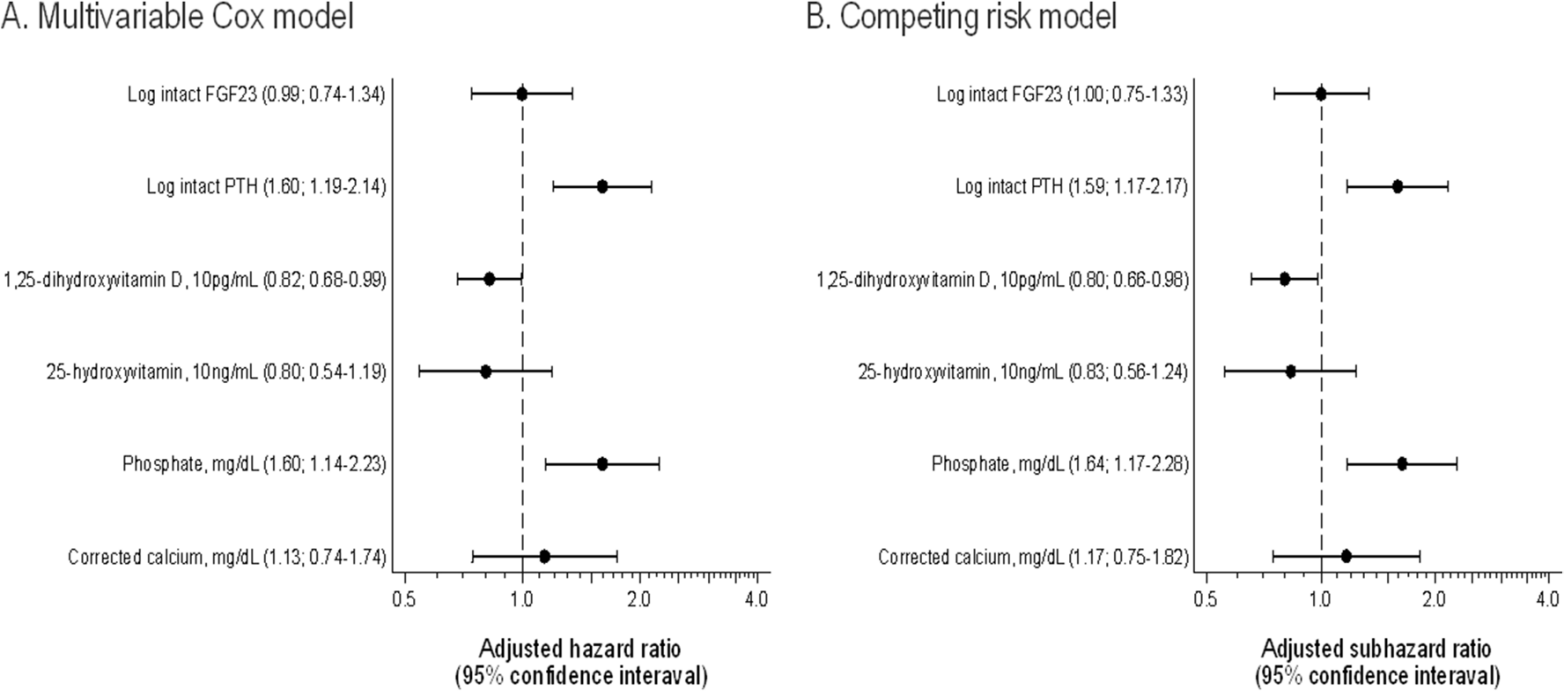
Forest plot depicting the effect of MBD parameters on outcome in a (**A**) multivariate Cox model and (**B**) competing risk model. Models were adjusted for age, sex, eGFR, urinary protein, donor type (living or deceased), donor age, transplantation vintage, and active vitamin D use. *PTH* parathyroid hormone; *FGF23* fibroblast growth factor 23; *eGFR* estimated glomerular filtration rate.

**Figure 2:**
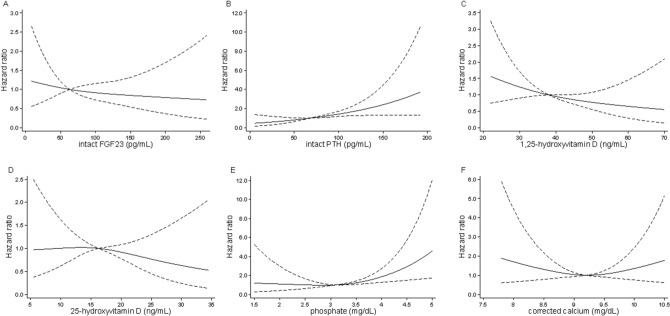
Associations between MBD parameters and renal outcomes. Models were adjusted for age, sex, eGFR, urinary protein, donor type (living or deceased), donor age, transplantation vintage, active vitamin D use, and MBD parameters (intact FGF23, intact PTH, 1,25-hydroxyvitamin D, 25-dihydroxyvitamin D, phosphate, and corrected calcium, as appropriate). Median values of MBD parameters were used as reference. The dashed lines indicate 95% confidence intervals. *PTH* parathyroid hormone; *FGF23* fibroblast growth factor 23; *eGFR* estimated glomerular filtration rate.

In the multivariate Cox model adjusting for clinically relevant factors, iPTH, phosphate, and 1,25D levels were still associated with the outcome, whereas FGF23 lost its predictive value (Table 2, Fig. 1). Figure 2 indicates RCS as describing associations between MBD parameters and primary outcome. The association of phosphate levels with the outcome was non-linear (P for linearity = 0.04).

### Interplays among MBD parameters and renal outcome

**Figure 3:**
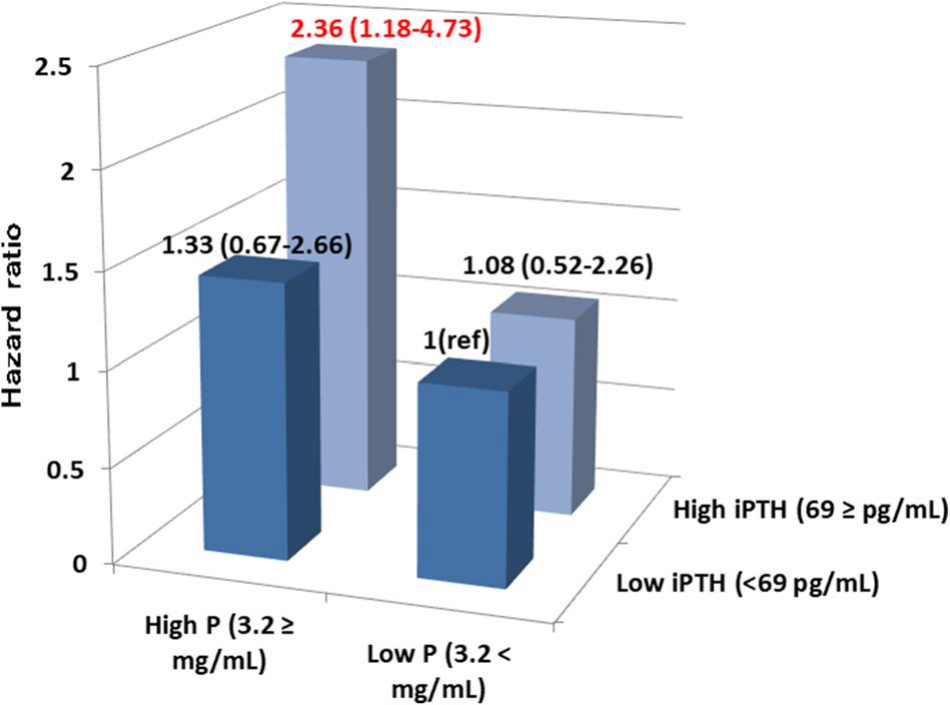
Adjusted hazard ratios (95% confidence interval) for renal outcomes across iPTH-phosphorus groups. Patients were stratified into four mutually exclusive groups according to the median values of iPTH and phosphorus levels. Models were adjusted for age, sex, eGFR, urinary protein, donor type (living or deceased), donor age, transplantation vintage, corrected calcium, intact FGF23, 1,25-dihydroxyvitamin D, 25-hydroxyvitamin D, and active vitamin D use. *PTH* parathyroid hormone; *FGF23* fibroblast growth factor 23; *eGFR* estimated glomerular filtration rate.

Since iPTH (not FGF23) was associated with renal phosphate wasting in KTRs 1 year after KTx^16^, we examined whether serum phosphate levels modified the relationship between iPTH and outcome, by including interaction terms in the multivariate Cox model. The interaction between iPTH and phosphate levels was significant (P for interaction = 0.06). Patients were stratified into four mutually exclusive groups according to the median values of iPTH and phosphate levels. In the presence of high phosphate levels, high iPTH was associated with an unfavorable renal outcome. However, in the absence of high phosphate levels, high iPTH did not have an impact (Fig. 3). Compared with the high iPTH/low phosphate group, the high iPTH/high phosphate group had higher TmP/GFR, longer transplant vintage, and comparable dialysis vintage (Supplementary Table S1).

**Figure 4:**
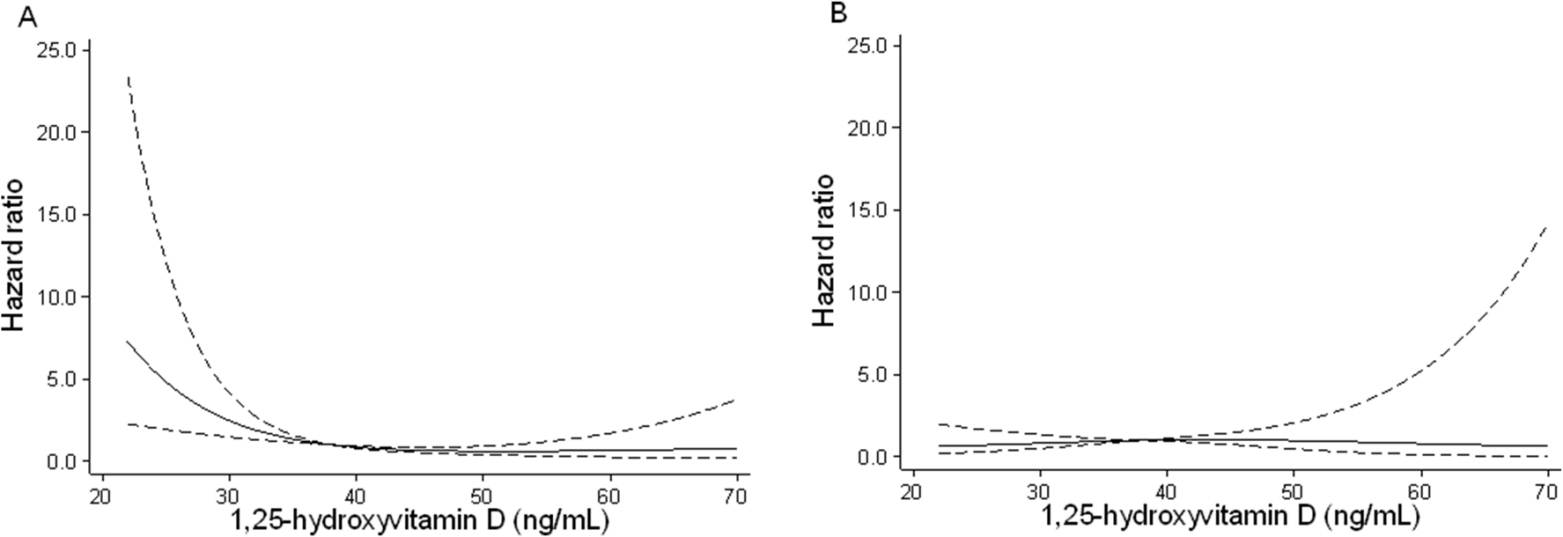
Associations of 1,25D levels with outcomes (**A**) without and (**B**) with active vitamin D. Models were adjusted for age, sex, eGFR, urinary protein, donor type (living or deceased), donor age, transplantation vintage, and MBD parameters (intact FGF23, intact PTH, corrected calcium, phosphate, and 25-dihydroxyvitamin D). Median values of 1.25D were used as reference. *PTH* parathyroid hormone; *FGF23* fibroblast growth factor 23; *eGFR* estimated glomerular filtration rate.

In addition, we also examined whether the use of active vitamin D modified the association between 1,25D levels and outcome. Since their interaction was significant (P for interaction = 0.05), KTRs were divided into two groups, i.e., with and without active vitamin D. Our results showed that poor 1,25D status predicted an unfavorable outcome only in KTRs not receiving active vitamin D (Fig. 4).

### Subgroup and sensitivity analyses

In subgroup analysis excluding patients with a history of parathyroidectomy (5.7%), the results remained noticeably unchanged (data not shown). Since we enrolled prevalent KTRs with a variety of durations after KTx and allograft function, we categorized patients according to transplantation vintage (over 10 years or not) and eGFR (over 45 mL/min/1.73 m^2^ or not) to confirm the robustness of our results. Despite wide confidence intervals due to low statistical power in subgroup analyses, point estimates were not substantially changed especially in iPTH and phosphate (Supplementary Table S2).

For sensitivity analyses, we employed a multivariate Cox regression with backward stepwise elimination of all variables in Table 1, with P-value ≧ 0.15 (the variables of iPTH, 1,25D, and phosphate levels were forcedly retained in the analysis), because of potential overfitting. As with the primary results, iPTH, phosphate, and 1,25D levels predicted the outcome (Supplementary Table S3). In addition, a competing risk analysis with death as a competing event and a competing risk analysis with all variables in Table 1 using a backward stepwise method with a P-value ≧ 0.10 yielded similar results to those generated using the Cox model (Fig. 1, Supplementary Table S4, S5).

## Discussion

In this prospective cohort of KTRs with a median follow-up over a period of 10 years, high iPTH, phosphate, and low 1,25D levels predicted poor renal outcome. In contrast, although FGF23 and 25D levels were associated with the outcome in univariate analyses, these associations lost significance following adjustment for other MBD parameters and clinically relevant factors. We also found two potentially important interactions between MBD-related factors: (1) phosphate levels modified the association of iPTH levels and outcome and (2) active vitamin D use modified the association of 1,25D levels with outcome.

In concordance with our results, several large cohorts^8–12^ revealed that post-transplant higher phosphate and iPTH levels were associated with unfavorable graft survival. A large study comprising 3,138 KTRs reported that a 1 mg/dL higher phosphate level was associated with 72% greater risk of graft failure^9^; however, this investigation did not account for important phosphaturic hormones (i.e., PTH and FGF23) or vitamin D (i.e., 25D and 1,25D). A post hoc Assessment of LEscol in Renal Transplantation Analysis (ALERT) trial demonstrated high iPTH and phosphate levels as being independently associated with death-censored graft loss; unfortunately, however, this study appears not to have measured FGF23, 1,25D, or 25D^10^. Meanwhile, Jeon et al.^8^ reported that serum phosphorus showed a U-shaped association with graft outcome; however, unmeasured 25D and 1,25D may explain the association between low phosphate levels and poor graft survival. To our knowledge, the current study is the first report to simultaneously evaluate a panel of six important MBD variables.

It is uncertain to how high iPTH and phosphate levels relate to allograft prognosis. The authors of previous studies speculated that these relationships can potentially be attributed to the direct and indirect effects of PTH on vasculature and so-called phosphate toxicity^9,10^. It is assumed that PTH directly stimulates aldosterone synthesis in the adrenal glands. On the contrary, aldosterone increases PTH secretion by binding to mineral corticoid receptors in the parathyroid gland^24^. This interplay between PTH and aldosterone may contribute to the development of allograft dysfunction. Nephrocalcinosis is a possible mediator linking high iPTH and poor graft outcome^6^. However, the effect size of iPTH remained substantially unchanged after adjustment for serum calcium and phosphate levels in our study (Table 2), suggesting that nephrocalcinosis is not present in the causal pathway between high iPTH and impaired graft survival. Regarding phosphate toxicity, much lower serum phosphate levels in KTRs compared with those in CKD patients^16^ may raise questions about this concept.

The observed interaction between iPTH and phosphate levels offers a different perspective. Given that PTH is a phosphaturic hormone, in the context of high PTH levels, low phosphate levels implied fair kidney responsiveness to PTH, as shown in significantly lower TmP/GFR as compared to those in the high iPTH/high phosphate group (Supplementary Table S3). The link between fair kidney responsiveness and favorable outcomes is compatible with a report that severe hypophosphatemia (defined as phosphate levels < 1.55 mg/dL) within 1 year after KTx (usually a period of high PTH) predicts favorable graft survival^11^. In addition, poor kidney responsiveness to PTH may explain the association between high iPTH and poor outcome in the whole analysis. Generally, PTH levels decrease rapidly within the first 12 months after KTx, followed by a gradual decline^6^. In the early post-transplant period, pre-transplant hyperparathyroidism contributes to inappropriate hypercalcemia and/or hypophosphatemia in the presence of improved kidney responsiveness to PTH. Thereafter, along with the loss of kidney responsiveness to PTH, an adaptive PTH elevation (de novo MBD) occurs. Considering that pre-transplant iPTH levels were not a prognostic factor for graft survival^25^, high iPTH originating from de novo MBD but not from carryover MBD may be linked to the unfavorable outcome via PT dysfunction. In line with our hypothesis, KTRs with high iPTH and high phosphate levels had two times longer median transplant vintage than those with high iPTH and low phosphate levels (Supplementary Table S1).

We did not observe an association between FGF23 levels and outcome. To date, two studies evaluating the association of FGF23 with renal prognosis in KTRs yielded conflicting results. Wolf et al.^12^ reported C-terminal FGF23 to be significantly associated with allograft loss in KTRs following adjustment for only 3 factors of eGFR, age, and gender. In contrast, a study conducted in France demonstrated that C-terminal FGF23 was not an independent predictor of graft loss ^13^. Potential explanations for the absence of the association of FGF23 with the outcome in our study include the following: (1) different assays for FGF23 measurement (intact vs. C-terminal); (2) long-term median follow-up (10.7 vs. 3.1 years^12^) as a prognostic value of FGF23 was attenuated with a long-term follow-up period^13,26^; and (3) low baseline eGFR (38 vs. 51 min/mL/1.73m^2^^12^), due to the attenuated association between FGF23 and renal outcome in advanced CKD^27^. Further studies are warranted to clarify the role of FGF23 on renal graft outcomes.

In our study, 1,25D (but not 25D) levels independently predicted the outcome, even after adjustment for clinically relevant factors such as eGFR and proteinuria. To date, few studies have investigated the link between vitamin D levels and graft failure. Bienaimé et al.^14^ reported neither 25D nor 1,25D levels at 3 months after KTx was associated with graft loss. Keyzer et al.^15^ showed that 1,25D, but not 25D, levels at least 1 year after KTx predicted graft failure in a univariate model, but they lost their predictive value following adjustment for renal function. This inconsistency may have been derived from differences in participant characteristics (i.e., race and transplant vintage), practice pattern (i.e., active and/or nutritional vitamin D use), and assays conducted for vitamin D. Two possible mechanisms may explain the association between low 1,25D levels and non-preferred renal outcomes. First, low 1,25D levels may be a proxy of impaired proximal tubular function. Second, the renoprotective effects of 1,25D may contribute to its association^28^. Moreover, active vitamin D use served as a modifier for the relationship between 1,25D levels and outcome. Only in KTRs not receiving active vitamin D, low 1,25D levels were associated with poor outcome. To date, a small observational study among KTRs^29^, as well as a propensity-matched study of CKD^30^, suggests that active vitamin D therapy improves renal outcomes. Future randomized interventional studies are therefore warranted to assess the effect of active vitamin D use on graft outcome.

The strengths of the current study include its prospective design with detailed baseline information and long-term follow-up period (over a period of 10 years), providing it with sufficient statistical power. We performed rigorous adjustments for a comprehensive panel of circulating MBD parameters. Several limitations of the research should also, however, be noted. As this was a single-center study, results are not generalizable to KTRs in other countries. Because of its observational nature, we were also unable to establish causality. The lack of quantitative urine protein excretion may result in residual confounding. Also, urinary tubular injury biomarkers such as neutrophil gelatinase-associated lipocalin and β2-microglobulin were unavailable. Besides, our population was heterogeneous especially regarding transplantation vintage and allograft function, however, stratified analyses yielded robust results.

In conclusion, iPTH, phosphate, and 1,25D, but not FGF23, levels predicted renal outcome in KTRs with grafts functioning at least 1 year after KTx. Since the association of high iPTH levels with poor outcome was not observed in KTRs with low phosphate levels, low phosphate levels in a high iPTH level setting were considered benign manifestations. Future prospective interventional studies are needed to confirm the benefits of active vitamin D use in KTRs with low 1,25D levels.

## Supplementary information

Supplementary file1.
