# Supplementary material for: Serum phosphate levels modify the impact of parathyroid hormone levels on renal outcomes in kidney transplant recipients
Source: Sci Rep. 2020 Aug 13;10:13766. doi: 10.1038/s41598-020-70709-4 (PMC7426845; doi:10.1038/s41598-020-70709-4)
Supplement: Supplementary file 1 — Supplementary file1. [file 41598_2020_70709_MOESM1_ESM.docx]

Title: Serum phosphate levels modify the impact of parathyroid hormone levels on renal outcomes in kidney transplant recipients

Yohei Doi, MD^1^, Takayuki Hamano, MD, PhD^2, 3^, Naotsugu Ichimaru, MD, PhD^4^, Kodo Tomida, MD, PhD^5^, Yoshitsugu Obi, MD, PhD^6^, Naohiko Fujii, MD, PhD^7^, Satoshi Yamaguchi, MD^1^, Tatsufumi Oka, MD^1^, Yusuke Sakaguchi, MD, PhD^2^, Isao Matsui, MD, PhD^1^, Jun-ya Kaimori, MD, PhD^1^, Toyofumi Abe, MD, PhD^8^, Ryoichi　Imamura, MD, PhD^8^, Shiro Takahara, MD, PhD^9^, Yoshiharu Tsubakihara, MD, PhD^10^, Norio Nonomura, MD, PhD^8^, and Yoshitaka Isaka, MD, PhD^1^

*1. Department of Nephrology, Osaka University Graduate School of Medicine, Suita, Japan*

*2. Department of Inter-Organ Communication Research in Kidney Disease, Osaka University Graduate School of Medicine, Suita, Japan*

*3. Department of Nephrology, Nagoya City University Graduate School of Medical Sciences, Nagoya, Japan*

*4. Department of Advanced Technology for Transplantation, Osaka University Graduate School of Medicine, Suita, Japan*

*5. Department of Nephrology, Yodogawa Christian Hospital, Osaka, Japan.*

*6. Division of Nephrology, University of Tennessee Health Science Center, Memphis, USA.*

*7. Department of Nephrology, Hyogo Prefectural Nishinomiya Hospital, Nishinomiya, Japan.*

*8. Department of Urology, Osaka University Graduate School of Medicine, Suita, Japan.*

*9. Department of Renal Transplantation, Kansai Medical Hospital, Toyonaka, Japan.*

*10. Graduate School of Health Care Sciences, Jikei Institute, Osaka, Japan*

Supplementary Table 1. Baseline characteristics of four groups categorized according to the median values of iPTH and phosphate levels^a^

|  | 1: Low iPTH-low phosphate  n = 68 | 2: Low iPTH-high phosphate n = 66 | 3: High iPTH-low phosphate  n = 66 | 4: High iPTH-high phosphate  n = 63 | P-value (3 vs 4)^b^ |
| --- | --- | --- | --- | --- | --- |
| Basic information |  |  |  |  |  |
| Age, years | 46 (38–59) | 46 (38–57) | 50 (40–60) | 54 (45–61) | 0.38 |
| Male sex, n (%) | 51 (75) | 37 (56) | 42 (64) | 33 (52) | 0.22 |
| BMI, kg/m^2^ | 21 (19–23) | 21 (19–24) | 22 (20–25) | 22 (20–24) | 0.71 |
| SBP, mmHg | 118 (111–126) | 121 (114–130) | 123 (119–130) | 125 (116–135) | 0.29 |
| Prior PTx, n (%) | 3 (4) | 5 (8) | 2 (3) | 5 (8) | 0.27 |
| Dialysis vintage, years | 1.8 (0.8–3.0) | 1.6 (0.7–5.0) | 3.4 (1.7–6.8) | 3.4 (0.9–6.8) | 0.41 |
| Transplant vintage, years | 11.9 (6.4–16.2) | 12.3 (5.6–16.9) | 6.4 (2.8–17.2) | 11.9 (4.2–19.1) | 0.04 |
| ABO incompatibility, n (%) | |  |  |  | 0.15 |
| Compatible | 54 (86) | 52 (87) | 47 (77) | 51 (88) |  |
| Incompatible | 9 (14) | 8 (13) | 14 (23) | 7 (12) |  |
| HLA mismatches (A+B+DR), n (%) | |  |  |  | 0.47 |
| 0 | 5 (8) | 6 (11) | 8 (14) | 6 (12) |  |
| 1–3 | 53 (86) | 47 (83) | 40 (69) | 40 (78) |  |
| 4–6 | 4 (7) | 4 (7) | 10 (17) | 5 (10) |  |
| Living donor, n (%) | 60 (88) | 54 (82) | 54 (82) | 49 (78) | 0.66 |
| Donor age, years | 50 (40–57) | 53 (43–59) | 54 (46–61) | 53 (40–61) | 0.37 |
| Medications, n (%) |  |  |  |  |  |
| Calcineurin inhibitor |  |  |  |  | 0.02 |
| Cyclosporine | 41 (60) | 39 (59) | 26 (40) | 37 (59) |  |
| Tacrolimus | 20 (29) | 22 (33) | 36 (55) | 19 (30) |  |
| None | 7 (10) | 5 (8) | 4 (6) | 7 (11) |  |
| Antimetabolic agents |  |  |  |  | 0.43 |
| Azathioprine | 17 (25) | 16 (24) | 8 (12) | 10 (16) |  |
| Mycophenolate mofetil | 25 (37) | 37 (56) | 44 (67) | 37 (59) |  |
| Mizoribine | 19 (28) | 5 (8) | 12 (18) | 10 (16) |  |
| None | 7 (10) | 8 (12) | 2 (3) | 6 (10) |  |
| Prednisolone, n (%) | 66 (97) | 65 (99) | 65 (99) | 60 (95) | 0.36 |
| Active vitamin D compounds, n (%) | 33 (49) | 39 (59) | 19 (30) | 25 (40) | 0.27 |
| CaCO_3_, n (%) | 0 (0) | 2 (3) | 0 (0) | 2 (3) | 0.24 |
| RAAS inhibitors, n (%) | 46 (68) | 48 (73) | 43 (65) | 51 (81) | 0.05 |
| Primary renal disease, n (%) | |  |  |  | 0.10 |
| Chronic glomerulonephritis | 37 (54) | 36 (55) | 35 (53) | 24 (38) |  |
| Diabetic nephropathy | 1 (2) | 4 (6) | 2 (3) | 6 (10) |  |
| Unknown | 17 (25) | 14 (21) | 19 (29) | 27 (43) |  |
| Others | 13 (19) | 12 (18) | 10 (15) | 6 (10) |  |
| Laboratory data |  |  |  |  |  |
| Hemoglobin, g/dL | 12.8 (11.8–13.9) | 11.8 (10.8–13.1) | 12.5 (11.6–13.9) | 11.7 (10.8–12.9) | <0.01 |
| Albumin, mg/dL | 4.3 (4.1–4.5) | 4.2 (4.1–4.4) | 4.3 (4.1–4.5) | 4.2 (4.0–4.4) | 0.04 |
| eGFR, mL/min per 1.73 m^2^ | 48 (35–58) | 35 (28–48) | 40 (33–54) | 33 (21–46) | <0.01 |
| Corrected calcium, mg/dL | 9.2 (8.9–9.5) | 9.2 (9.0–9.6) | 9.4 (9.0–9.9) | 9.1 (8.8–9.3) | <0.01 |
| Phosphate, mg/dL | 2.8 (2.6–3.0) | 3.5 (3.3–3.7) | 2.7 (2.5–2.9) | 3.7 (3.4–4.2) | <0.01 |
| 25D, ng/mL | 18 (14–24) | 18 (14–21) | 16 (12–20) | 15 (10–20) | 0.15 |
| 1,25D, pg/mL | 40 (30–49) | 32 (23–41) | 46 (33–63) | 36 (25–51) | <0.01 |
| Intact PTH, pg/mL | 50 (40–58) | 46 (35–58) | 102 (83–141) | 102 (84–141) | 0.12 |
| Intact FGF23, pg/mL | 52 (37–82) | 80 (56–185) | 53 (39–80) | 75 (46–161) | 0.13 |
| Urinary protein, n (%) |  |  |  |  | 0.45 |
| - | 45 (66) | 36 (55) | 32 (49) | 24 (38) |  |
| 1+ | 19 (28) | 24 (36) | 27 (41) | 29 (46) |  |
| 2–3+ | 4 (6) | 6 (9) | 7 (11) | 10 (16) |  |
| TmP/GFR, mg/dL | 2.2 (1.8-2.4) | 2.7 (2.5-2.9) | 1.8 (1.6-2.1) | 2.6 (2.4-2.9) | <0.01 |

^a^The values are presented as median (interquartile range) or number (%).

^b^Differences between the high iPTH–low phosphate group and high iPTH–high phosphate group were tested using Fisher's exact test and Welch's t-test, as appropriate.

Abbreviations: BMI, body mass index; SBP, systolic blood pressure; PTx, parathyroidectomy; RAAS, renin–angiotensin–aldosterone system; eGFR, estimated glomerular filtration rate; 25D, 25-hydroxyvitamin D; 1,25D, 1,25-dihydroxyvitamin D; PTH, parathyroid hormone; FGF23, fibroblast growth factor 23; TmP/GFR, tubular maximum reabsorption rate of phosphate to the glomerular filtration rate.

Supplementary Table 2. Adjusted hazard ratios (95% confidence interval) for renal outcomes according to baseline transplantation vintage and eGFR

|  | Whole cohort | Tvin>10 | Tvin<=10 | eGFR<45 | eGFR>=45 |
| --- | --- | --- | --- | --- | --- |
| Log intact PTH | 1.60 (1.19-2.14) | 1.49 (0.89-2.50) | 1.95 (1.26-3.02) | 1.43 (1.03-1.98) | 1.47 (0.65-3.33) |
| Phosphate, mg/dL | 1.60 (1.14-2.23) | 1.77 (0.96-3.24) | 1.67 (1.06-2.63) | 1.54 (1.02-2.31) | 1.62 (0.57-4.61) |
| 1,25D, 10pg/mL | 0.82 (0.68-0.99) | 0.76 (0.56-1.05) | 0.91 (0.70-1.19) | 1.00 (0.80-1.25) | 0.58 (0.37-0.92) |

Patients were stratified into 2 groups according to transplantation vintage and eGFR. We found no significant interaction between MBD parameters (iPTH, Phosphate, and 1,25D) with transplantation vintage or eGFR (all p-values > 0.4). Models were adjusted for age, sex, eGFR, urinary protein, donor type (living or deceased), donor age, transplantation vintage, corrected calcium, intact FGF23, 1,25-dihydroxyvitamin D, 25-hydroxyvitamin D, and active vitamin D use. Abbreviations: PTH, parathyroid hormone; eGFR, estimated glomerular filtration rate; 1,25D, 1,25-dihydroxyvitamin D

Supplementary Table 3. Results of a multivariable Cox model using a backward stepwise method

| variables | Multivariable Cox Proportional Model | | |
| --- | --- | --- | --- |
|  | Hazard Ratio | 95% CI | P Value |
| Urine protein - | ref |  |  |
| 1+ | 2.57 | 1.58-4.16 | <0.01 |
| 2–3+ | 2.98 | 1.49-5.97 | <0.01 |
| Phosphate, mg/dL | 1.55 | 1.15-2.08 | <0.01 |
| 1.25D, 10pg/mL | 0.81 | 0.69-0.95 | 0.01 |
| Log intact PTH | 1.52 | 1.17-1.99 | <0.01 |
| Hemoglobin, g/dL | 0.86 | 0.74-1.01 | 0.07 |
| Log transplant vintage | 1.33 | 1.01-1.75 | 0.04 |
| Azathioprine use | 0.45 | 0.25-0.83 | 0.01 |

Abbreviations: 95% CI, 95% confidence interval; 1,25D, 1,25-dihydroxyvitamin D; PTH, parathyroid hormone

Supplementary Table 4. Results of a competing risk model

| variables | Competing Risk Model | | |
| --- | --- | --- | --- |
|  | Subhazard Ratio | 95% CI | P Value |
| Age, years | 0.99 | 0.97-1.01 | 0.32 |
| Male sex | 1.12 | 0.67-1.87 | 0.67 |
| eGFR, mL/min/1.73m^2^ | 0.99 | 0.96-1.02 | 0.49 |
| Urine protein - | ref |  |  |
| 1+ | 2.85 | 1.68-4.85 | <0.01 |
| 2-3+ | 2.96 | 1.36-6.42 | 0.01 |
| Corrected calcium, mg/dL | 1.17 | 0.75-1.82 | 0.50 |
| Phosphate, mg/dL | 1.64 | 1.17-2.28 | <0.01 |
| 25D, 10ng/mL | 0.83 | 0.56-1.24 | 0.37 |
| 1.25D, 10pg/mL | 0.80 | 0.66-0.98 | 0.03 |
| Log intact PTH | 1.59 | 1.17-2.17 | <0.01 |
| Log intact FGF23 | 1.00 | 0.75-1.33 | 0.99 |
| Active vitamin D compounds | 0.87 | 0.52-1.47 | 0.60 |
| Living donor | 1.03 | 0.57-1.88 | 0.91 |
| Donor age, year | 1.00 | 0.98-1.02 | 0.92 |
| Log transplant vintage | 1.31 | 0.94-1.81 | 0.11 |

Abbreviations: 95% CI, 95% confidence interval; GFR, estimated glomerular filtration rate; 25D, 25-hydroxyvitamin D; 1,25D, 1,25-dihydroxyvitamin D; PTH, parathyroid hormone; FGF23, fibroblast growth factor 23

Supplementary Table 5. Results of a competing risk model using a backward stepwise method

| variables | Competing Risk Model | | |
| --- | --- | --- | --- |
|  | Subhazard Ratio | 95% CI | P Value |
| Urine protein - | ref |  |  |
| 1+ | 2.74 | 1.67-4.49 | <0.01 |
| 2–3+ | 2.78 | 1.30-5.98 | 0.01 |
| Phosphate, mg/dL | 1.62 | 1.19-2.22 | <0.01 |
| 1.25D, 10pg/mL | 0.76 | 0.64-0.89 | <0.01 |
| Log intact PTH | 1.73 | 1.27-2.35 | <0.01 |
| Mizoribine use | 0.47 | 0.25-0.87 | 0.02 |
| Log transplant vintage | 1.44 | 1.10-1.88 | 0.01 |
| Azathioprine use | 0.40 | 0.22-0.73 | <0.01 |

Abbreviations: 95% CI, 95% confidence interval; 1,25D, 1,25-dihydroxyvitamin D; PTH, parathyroid hormone
